# Supplementary material for: Determination of d-Cycloserine Impurities in Pharmaceutical Dosage Forms: Comparison of the International Pharmacopoeia HPLC–UV Method and the DOSY NMR Method
Source: Molecules. 2020 Apr 7;25(7):1684. doi: 10.3390/molecules25071684 (PMC7181288; doi:10.3390/molecules25071684)
Supplement: Supplementary file 1 [file molecules-25-01684-s001.pdf]

Supplementary Materials

Determination of D-cycloserine Impurities in Pharmaceutical Dosage Forms: Comparison of The International Pharmacopoeia HPLC-UV Method and DOSY NMR Method

Damjan Makuc[1,#], Živa Švab[2,5,#,$], Katerina Naumoska[3], Janez Plavec[1,2,4] and Zdenko Časar[5,6,*]

1 Slovenian NMR Centre, National Institute of Chemistry, Hajdrihova 19, SI-1000 Ljubljana, Slovenia; damjan.makuc@ki.si, janez.plavec@ki.si
2 University of Ljubljana, Faculty of Chemistry and Chemical Technology, Večna pot 113, SI-1001 Ljubljana, Slovenia; janez.plavec@fkkt.uni-lj.si
3 Department of Food Chemistry, National Institute of Chemistry, Hajdrihova 19, SI-1000 Ljubljana, Slovenia; katerina.naumoska@ki.si
4 EN-FIST Centre of Excellence, Trg Osvobodilne fronte 13, SI-1000 Ljubljana, Slovenia; janez.plavec@enfist.si
5 Lek Pharmaceuticals d.d., Sandoz Development Center Slovenia, Verovškova ulica 57, SI-1526 Ljubljana, Slovenia
6 University of Ljubljana, Faculty of Pharmacy, Aškerčeva cesta 7, SI-1000, Ljubljana, Slovenia
\# These authors contributed equally
$ Current position at: Biotechnology Development Unit, International Institute for Genetic Engineering and Biotechnology, Trieste, Italy; ziva.marsetic@icgeb.org
* Correspondence: zdenko.casar@sandoz.com or zdenko.casar@ffa.uni-lj.si; Tel.: +386-1-5802079; Fax: +386-1-5683517

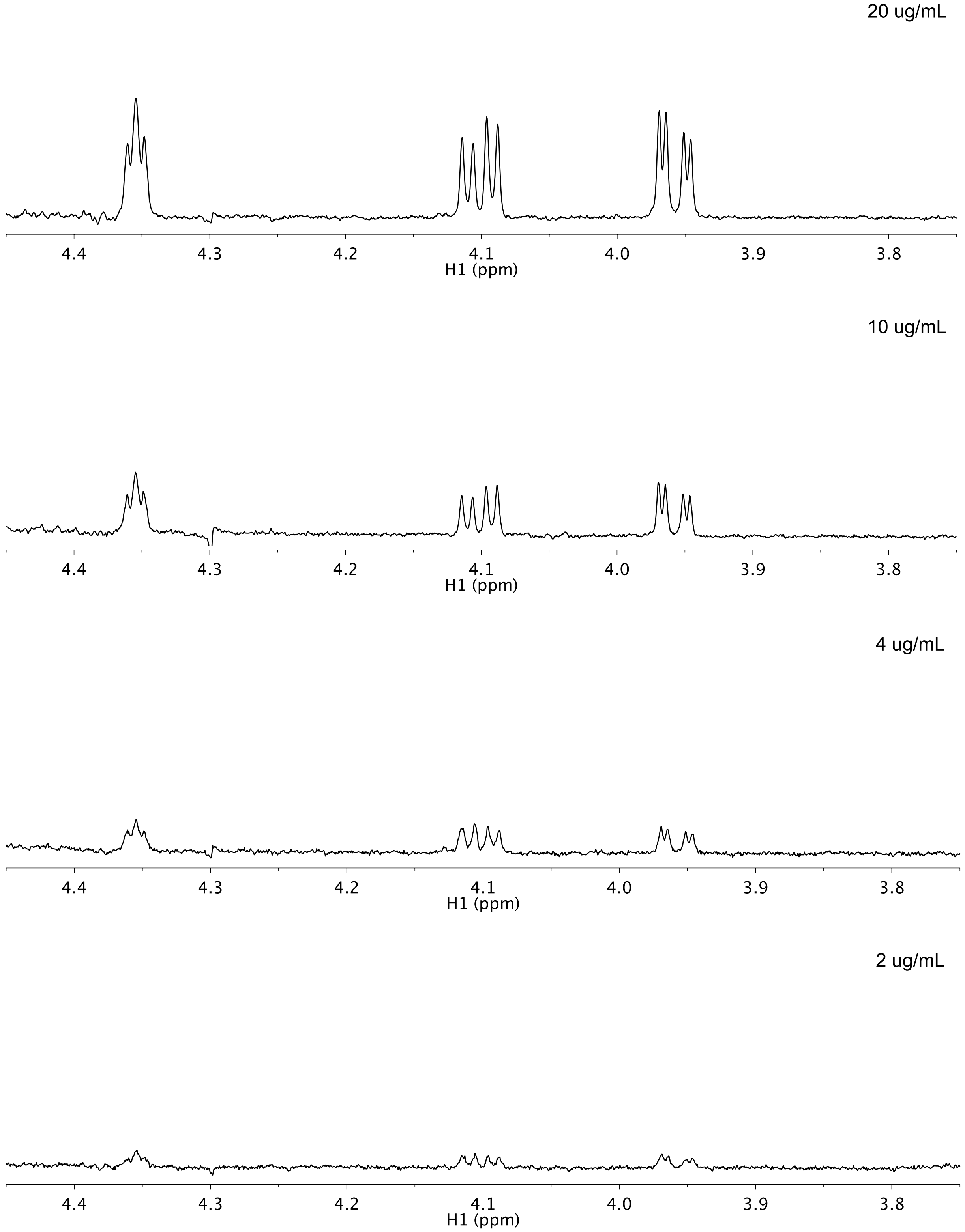

Figure S1: ^{1}H NMR spectra of cycloserine dimer in D_2O at 25 °C, concentrations between 2 and 20 μg mL^{-1}.

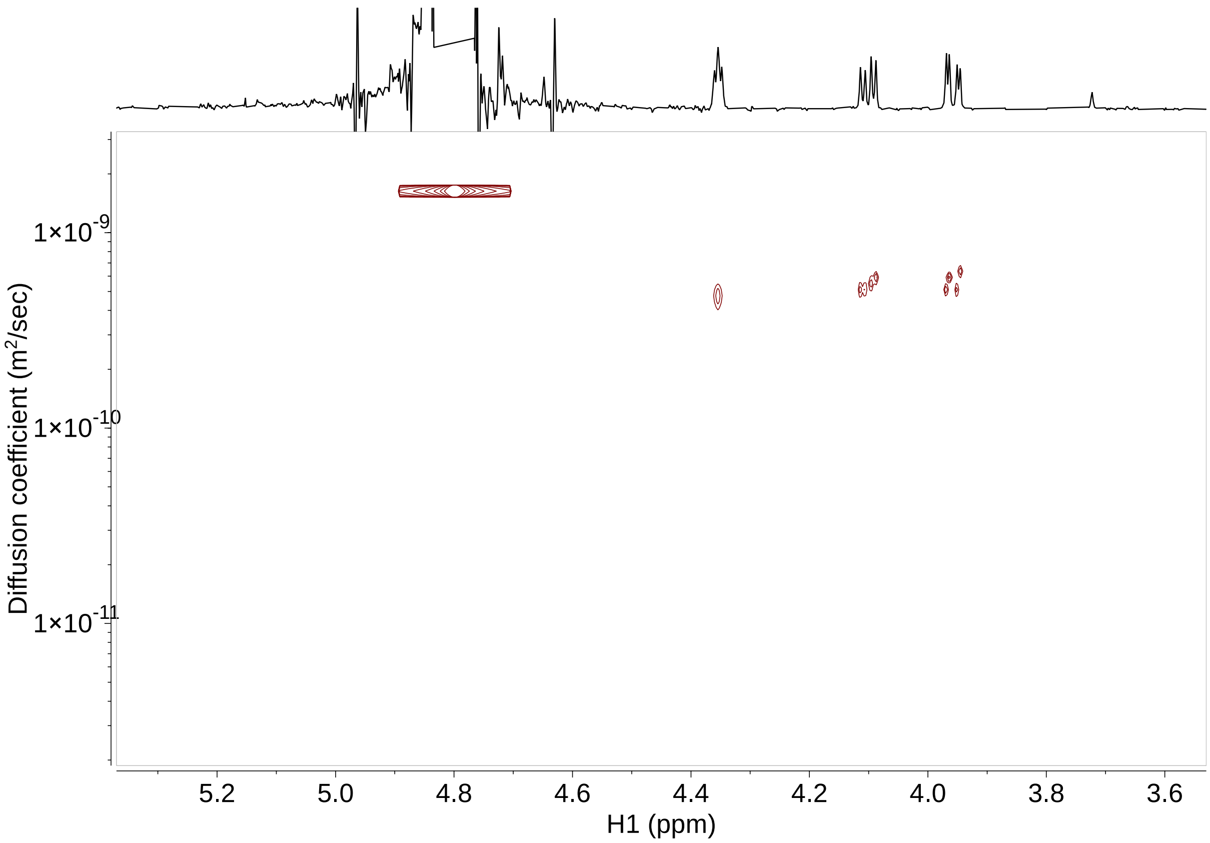

Figure S2. ^{1}H NMR (top) and ^{1}H DOSY NMR spectra (bottom) of cycloserine dimer at concentration 20 μg mL^{-1}.

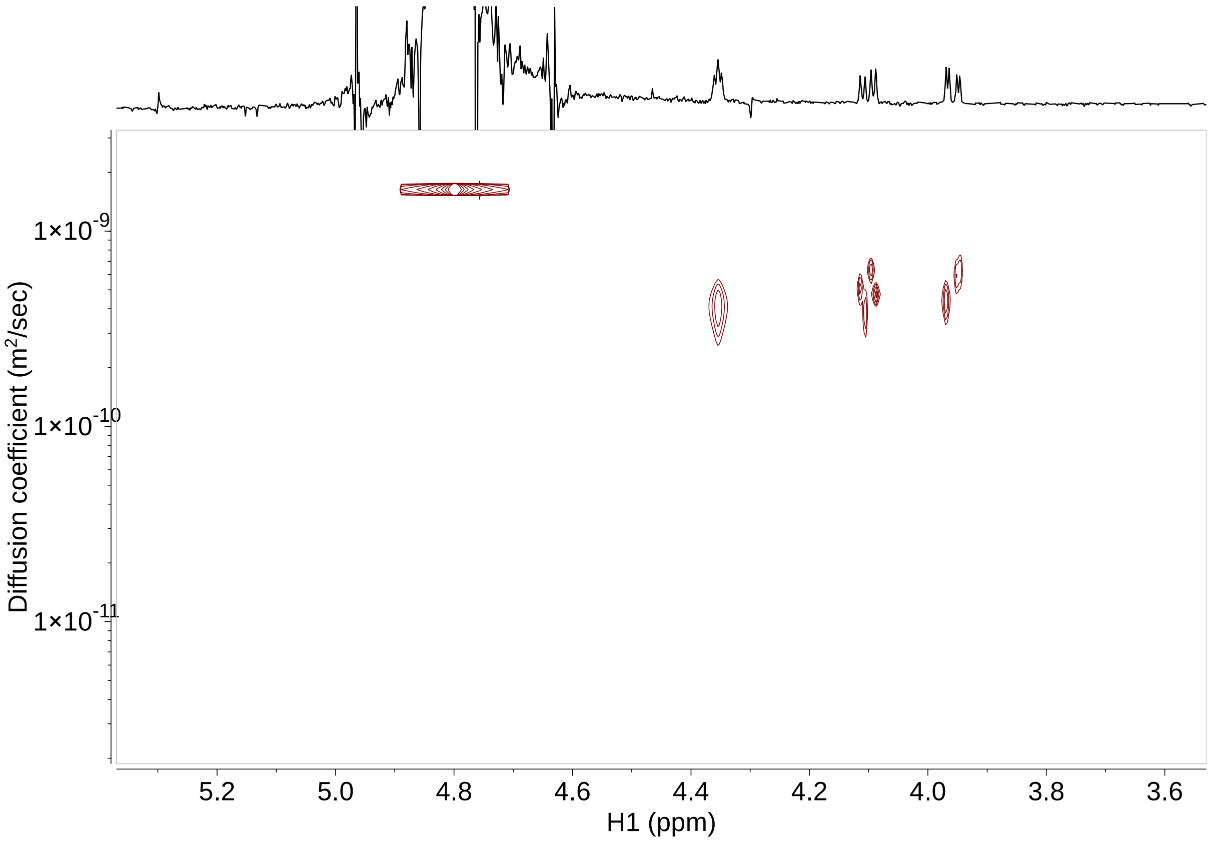

Figure S3. ^{1}H NMR (top) and ^{1}H DOSY NMR spectra (bottom) of cycloserine dimer at concentration 10 μg mL^{-1}.

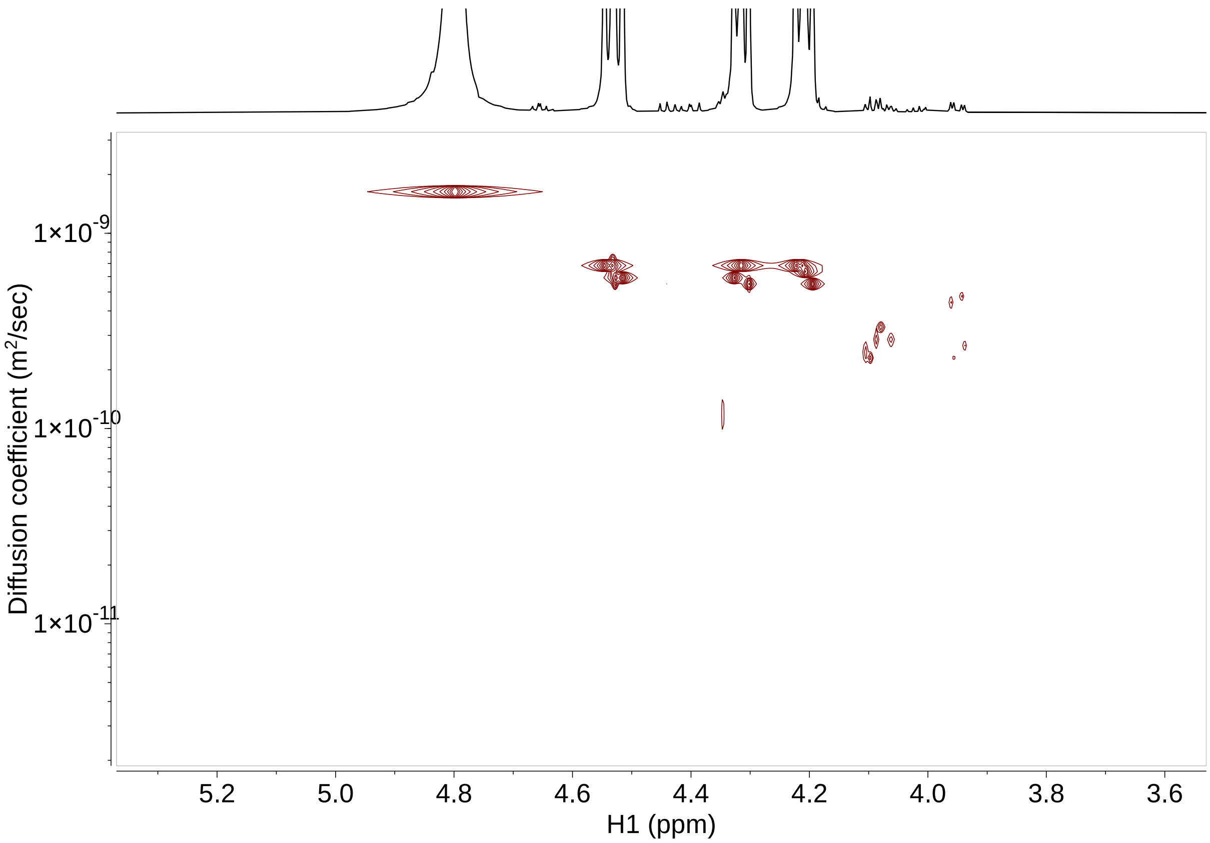

Figure S4. ^{1}H NMR (top) and ^{1}H DOSY NMR spectra (bottom) of D-cycloserine and cycloserine dimer within the quantification limit (0.05% relative to the concentration of D-cycloserine in the sample solution). Diffusion constant (D) is reported in $m^2s^{-1} \times 10^{-10}$.

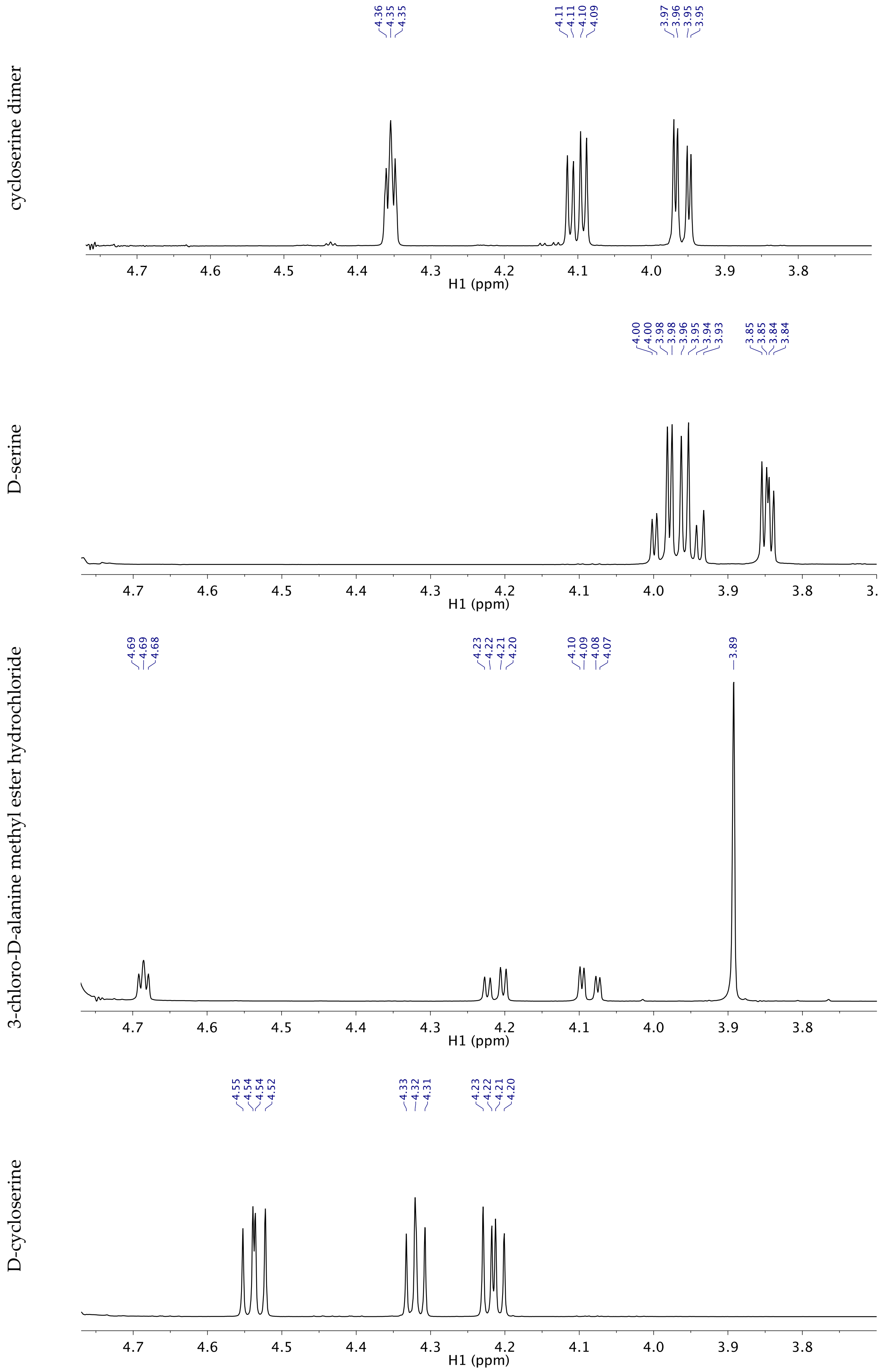

Figure S5. ^{1}H NMR spectra of D-cycloserine and impurities in D_2O at 25 °C.

Table S1. NMR stability test for D-cycloserine in sample solution.[1]

t (min)	m_x (mg)	I_x	I_{RS}	N_x	N_{RS}	P_x (%)
0	2.090	0.473	2.000	1	2	**91.4**
30	2.090	0.466	2.000	1	2	**90.1**
60	2.090	0.463	2.000	1	2	**89.5**

[1] M_x = 102.09 $gmol^{-1}$, m_{RS} = 4.594 mg, M_{RS} = 116.07 $gmol^{-1}$, P_{RS} = 99.98.

Table S2. NMR stability test for cycloserine dimer in sample solution.[1]

t (min)	m_x (mg)	I_x	I_{RS}	N_x	N_{RS}	P_x (%)
0	1.780	0.445	2.000	2	2	**101.0**
30	1.780	0.438	2.000	2	2	**99.4**
60	1.780	0.434	2.000	2	2	**98.5**

[1] M_x = 204.20 $gmol^{-1}$, m_{RS} = 4.594 mg, M_{RS} = 116.07 $gmol^{-1}$, P_{RS} = 99.98.

Table S3. NMR stability test for 3-chloro-D-alanine methyl ester HCl in sample solution.[1]

t (min)	m_x (mg)	I_x	I_{RS}	N_x	N_{RS}	P_x (%)
0	2.161	0.939	2.000	3	2	**99.7**
30	2.161	0.941	2.000	3	2	**100.0**
60	2.161	0.936	2.000	3	2	**99.4**

[1] M_x = 174.03 $gmol^{-1}$, m_{RS} = 4.594 mg, M_{RS} = 116.07 $gmol^{-1}$, P_{RS} = 99.98.

Table S4. NMR stability test for D-serine in sample solution.[1]

t (min)	m_x (mg)	I_x	I_{RS}	N_x	N_{RS}	P_x (%)
0	5.628	1.365	2.000	1	2	**100.9**
30	5.628	1.370	2.000	1	2	**101.2**
60	5.628	1.367	2.000	1	2	**101.0**

[1] M_x = 105.09 $gmol^{-1}$, m_{RS} = 4.594 mg, M_{RS} = 116.07 $gmol^{-1}$, P_{RS} = 99.98.

The stability of solution was tested over period of 0, 30 and 60 minutes. Results in Table S1 show that content (purity) of D-cycloserine dropped almost 2.0 % in 60 minutes. In addition, content of cycloserine dimer dropped 2.5 %. The other two analytes (3-chloro-D-alanine methyl ester HCl and D-serine) showed stability over the period of 1 hour. Based on stability test, all sample solution used for quantitative NMR measurements were freshly prepared and sample were dissolved just before acquisition of NMR data.

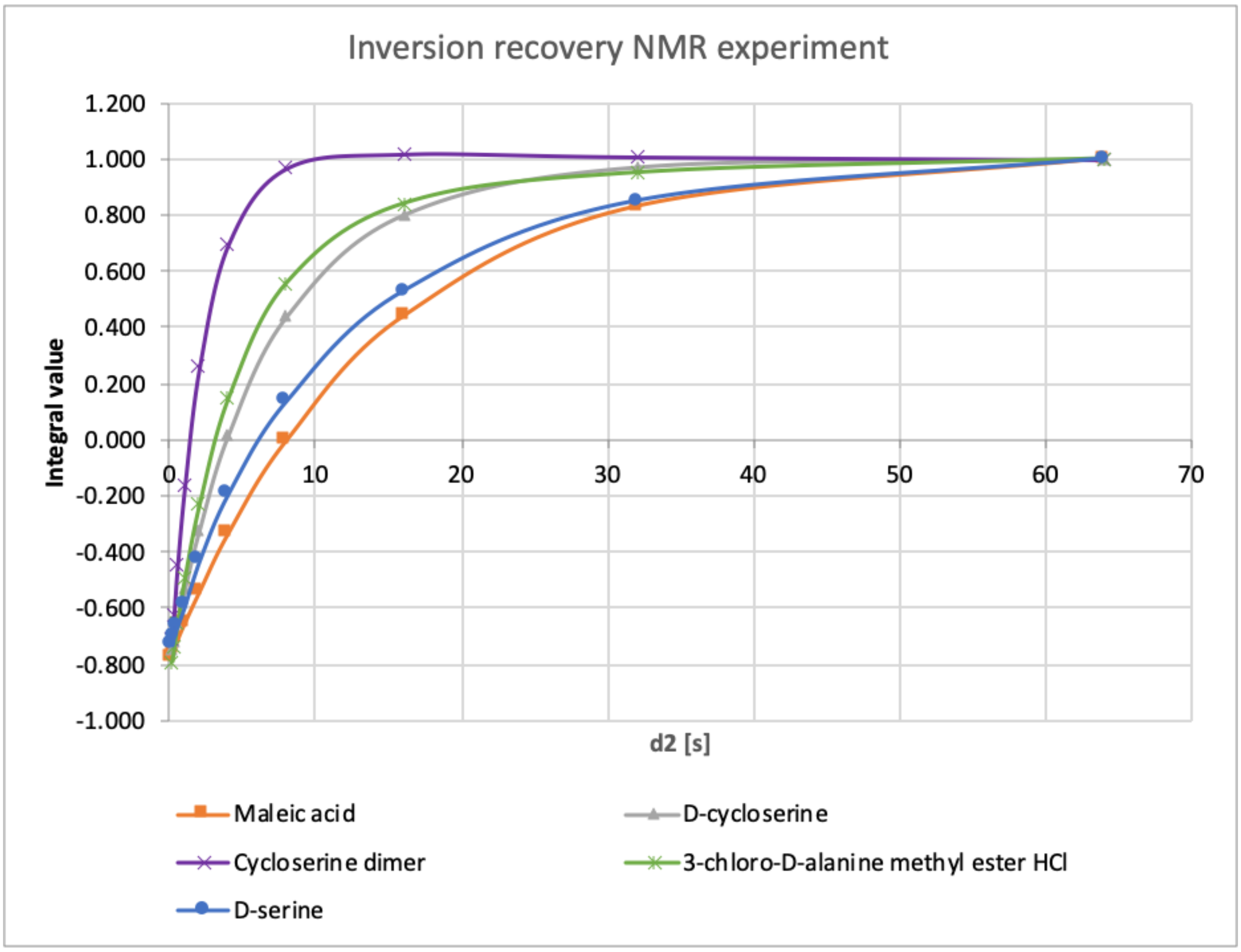

Figure S6. T_1 relaxation times were determined using the inversion recovery experiment operating on the basis of a 180° pulse, followed by a 90° pulse after a variable delay d2. The following T_1 relaxation times were determined for ^1H signals that were used for quantitative analysis: 5.60 s (D-cycloserine), 1.99 s (cycloserine dimer), 4.62 s (3-chloro-D-alanine methyl ester HCl), 9.09 s (D-serine) and 11.54 s (maleic acid). Relaxation delay d1=60 s (at least 5 x the longest resulting T_1) was used for all quantitative NMR measurements in the current study.

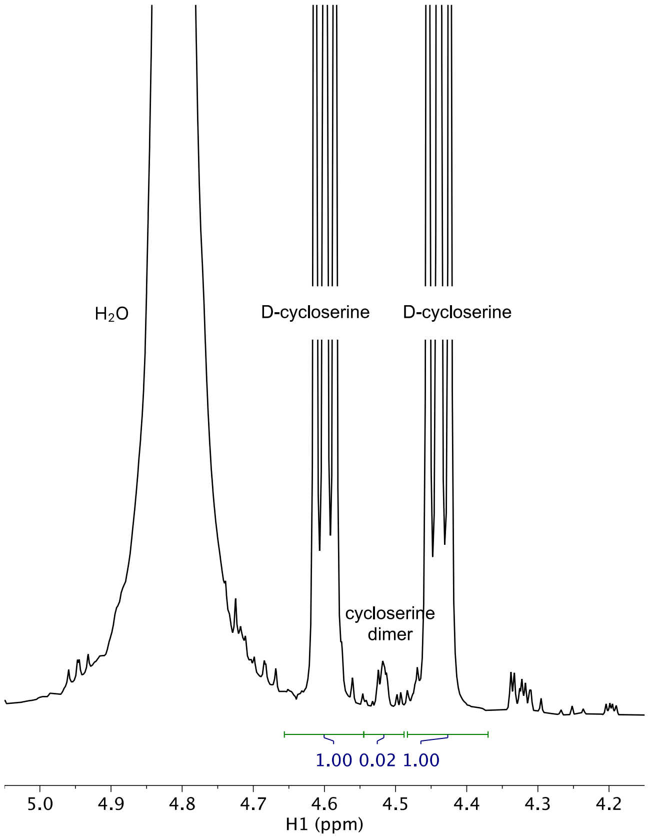

Figure S7. ^{1}H NMR spectrum were used to establish concentration of cycloserine dimer (0.1 mg mL^{-1}) in sample solution, which corresponds to 2% of D-cycloserine and is above specification limit ^{1}H NMR spectra of D-cycloserine and impurities in D_2O at 25 °C.
